# Voxel-based morphometry in single subjects without a scanner-specific normal database using a convolutional neural network

**DOI:** 10.1007/s00330-023-10356-1

**Published:** 2023-11-09

**Authors:** Julia Krüger, Roland Opfer, Lothar Spies, Dennis Hedderich, Ralph Buchert

**Affiliations:** 1 jung diagnostics GmbH, Hamburg, Germany; 2 Department of Neuroradiology, Klinikum rechts der Isar, Technical University of Munich, Munich, Germany; 3 Department of Diagnostic and Interventional Radiology and Nuclear Medicine, University Medical Center Hamburg-Eppendorf, Martinistr. 52, 20246 Hamburg, Germany

**Keywords:** Magnetic resonance imaging, Brain mapping, Alzheimer disease, Deep learning, Neural networks (computer)

## Abstract

**Objectives:**

Reliable detection of disease-specific atrophy in individual T1w-MRI by voxel-based morphometry (VBM) requires scanner-specific normal databases (NDB), which often are not available. The aim of this retrospective study was to design, train, and test a deep convolutional neural network (CNN) for single-subject VBM without the need for a NDB (CNN-VBM).

**Materials and methods:**

The training dataset comprised 8945 T1w scans from 65 different scanners. The gold standard VBM maps were obtained by conventional VBM with a scanner-specific NDB for each of the 65 scanners. CNN-VBM was tested in an independent dataset comprising healthy controls (*n* = 37) and subjects with Alzheimer’s disease (AD, *n* = 51) or frontotemporal lobar degeneration (FTLD, *n* = 30). A scanner-specific NDB for the generation of the gold standard VBM maps was available also for the test set. The technical performance of CNN-VBM was characterized by the Dice coefficient of CNN-VBM maps relative to VBM maps from scanner-specific VBM. For clinical testing, VBM maps were categorized visually according to the clinical diagnoses in the test set by two independent readers, separately for both VBM methods.

**Results:**

The VBM maps from CNN-VBM were similar to the scanner-specific VBM maps (median Dice coefficient 0.85, interquartile range [0.81, 0.90]). Overall accuracy of the visual categorization of the VBM maps for the detection of AD or FTLD was 89.8% for CNN-VBM and 89.0% for scanner-specific VBM.

**Conclusion:**

CNN-VBM without NDB provides a similar performance in the detection of AD- and FTLD-specific atrophy as conventional VBM.

**Clinical relevance statement:**

A deep convolutional neural network for voxel-based morphometry eliminates the need of scanner-specific normal databases without relevant performance loss and, therefore, could pave the way for the widespread clinical use of voxel-based morphometry to support the diagnosis of neurodegenerative diseases.

**Key Points:**

• *The need of normal databases is a barrier for widespread use of voxel-based brain morphometry.*

• *A convolutional neural network achieved a similar performance for detection of atrophy than conventional voxel-based morphometry.*

• *Convolutional neural networks can pave the way for widespread clinical use of voxel-based morphometry.*

**Graphical abstract:**

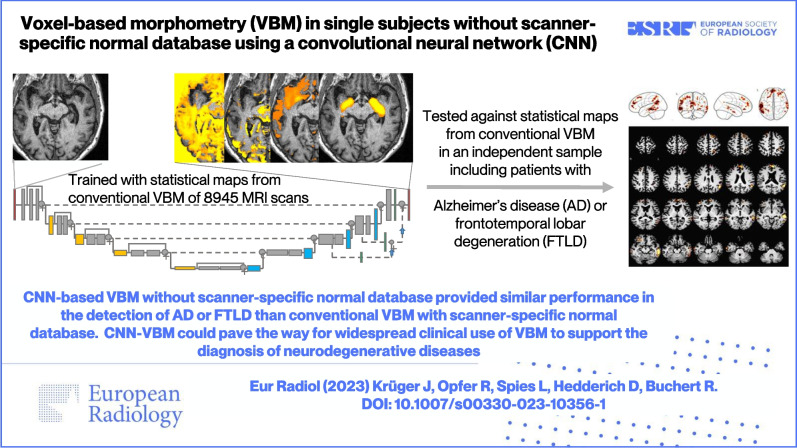

**Supplementary Information:**

The online version contains supplementary material available at 10.1007/s00330-023-10356-1.

## Introduction

Voxel-based morphometry (VBM) is a powerful technique of computational neuroanatomy based on brain MRI. It allows fully automatic, reader-independent identification of regional alterations of tissue concentrations throughout the whole brain without a priori hypotheses [1–6]. The output of the VBM is a map (VBM map) specifying the statistical significance of the tested effect on tissue concentration on the voxel level, e.g., regional gray matter (GM) loss in a patient or a group of patients compared to healthy controls.

VBM has been identified as an imaging biomarker to support the diagnosis and differential diagnosis of Alzheimer’s disease (AD) and other neurodegenerative disorders [7–9], for the identification of structural correlates of specific symptoms/syndromes [10–13], the prediction of cognitive decline [14], detection of brain structural changes associated with the exposure to potentially harmful substances [15], and brain involvement in non-neurological/non-psychiatric diseases [16, 17].

Reader-independent detection (or exclusion) of disease-specific atrophy patterns in the brain MRI of individual subjects has high potential to support diagnostics in clinical routine. Single-subject VBM, that compares the MRI scan of a single patient to a database of normal MRI scans, has proven promising for this purpose [18–22]. Hedderich and co-workers recently demonstrated that supporting visual analysis of brain MRI by single-subject VBM improves between-rater agreement and accuracy of MRI-based diagnosis and differential diagnosis of AD and frontotemporal lobar degeneration (FTLD) [22].

MRI-based volumetry including VBM is sensitive to the MRI scanner platform and to details of the acquisition sequence [23–28]. Thus, to achieve clinically useful sensitivity at low risk of false-positive findings, single-subject VBM requires a normal database (NDB) of MRI scans from control subjects acquired with the same MRI scanner and with exactly the same acquisition sequence as the individual scan to be analyzed. A scanner- and sequence-specific NDB consisting of 20–30 controls can be used, but NDB with two to three times larger size might provide better sensitivity [29] and/or better specificity [30]. The need for a sufficiently large scanner- and sequence-specific NDB (that has to be replaced after each relevant hardware and/or software update of the MRI scanner) is a major barrier for widespread clinical use of single-subject VBM.

This study trained and tested a convolutional neural network (CNN) for single-subject VBM without a NDB. For comparison, conventional VBM was tested with a non-scanner-specific NDB comprising normal scans from numerous different MRI scanners, which might be a rather unbiased conventional approach.

## Materials and methods

The MRI data of the training set and of the multiple-scanner NDB had been transferred to jung diagnostics GmbH under the terms and conditions of the European General Data Protection Regulation for remote image analysis. Subsequently, the data had been anonymized. The need for written informed consent for the retrospective use of the anonymized data in the present study was waived by the ethics review board of the General Medical Council of the state of Hamburg, Germany.

The MRI data of the test set were included retrospectively from previous studies [22, 31]. The use of the data for retrospective analyses was approved by the ethics committee of the Technical University of Munich (reference number 176/18s). Written informed consent was waived due to the retrospective nature of the analyses.

All procedures performed in this study were in accordance with the ethical standards of these ethics review boards and with the 1964 Helsinki Declaration and its later amendments.

All MR images included in this study had been acquired with the sequences for 3D gradient-echo T1w imaging of the brain provided by the scanner manufacturers. A summary of the datasets is given in Table 1.

**Table 1:** Training and test dataset

Dataset	Number of subjects	Diagnoses	Number of different scanners	Scanner manufacturers	Age [years] (mean ± std, range)
Training	8945	Unknown	65	Siemens: 44 Philips: 15 GE: 6	59.5 ± 17.5 15–99
Test	118	Healthy control: 37 Mild cognitive impairment due to Alzheimer’s disease: 22 Alzheimer’s disease dementia: 18 Posterior cortical atrophy: 11 Behavioral variant frontotemporal dementia: 20 Semantic variant primary progressive aphasia: 10	1	Siemens (Biograph PET-MRI)	63.4 ± 9.8 40–82
Multiple-scanner normal database	136	Normal T1w scan according to visual inspection	136	Siemens: 86 Philips: 34 GE: 4 Toshiba: 2	64.5 ± 8.2 45–83

### Training dataset

The training dataset comprised 8945 consecutive clinical T1w-MRI scans from 8945 different patients acquired with 65 different MRI scanners for various indications (Table 1). No eligibility criteria were applied to guarantee that the training set covered the whole range of T1w-MRI encountered in VBM in clinical routine. A separate scanner- and sequence-specific NDB of 25–120 MRI scans was available for each of the 65 scanners (total number of scans in the 65 NDB: 2150).

### Test dataset

The independent test dataset comprising T1w-MRI from 118 subjects was acquired with a hybrid PET-MRI scanner (Siemens Biograph MR, Siemens) that was not among the 65 scanners of the training dataset. The test dataset comprised 51 patients with AD (22 with mild cognitive impairment due to AD, 18 with typical AD dementia, 11 with posterior cortical atrophy), 30 patients with FTLD (20 with behavioral frontotemporal dementia, 10 with semantic primary progressive aphasia), and 37 healthy controls (Table 1). The ground truth diagnoses were established by experts based on biomarkers (FDG-PET, amyloid PET, cerebrospinal fluid), clinical examination, neuropsychological testing, and clinical follow-up.

Twenty-six healthy controls from the test dataset were used as scanner- and sequence-specific NDB for the test dataset. These healthy controls were not removed from the test dataset in order to avoid strong imbalance between patients with neurodegenerative disease and normal subjects in the test dataset.

### CNN-based VBM

The aim was to train a 3-dimensional (3D) CNN to generate full (non-thresholded) VBM maps in anatomical patient space without the need of a NDB (CNN-VBM).

Training data were prepared as follows. First, conventional single-subject VBM was applied to each of the 8945 MRI scans in the training dataset with the corresponding scanner- and sequence-specific NDB as reference (scanner-specific VBM). The SPM12 software package was used for this purpose (“Conventional single-subject VBM” in the supplementary material and Supplementary Figure 1). The resulting scanner-specific VBM maps were warped from template space to the anatomical space of the individual subjects by using the inverse of the individual transformations from patient to template space estimated during conventional VBM.

**Fig. 1:**
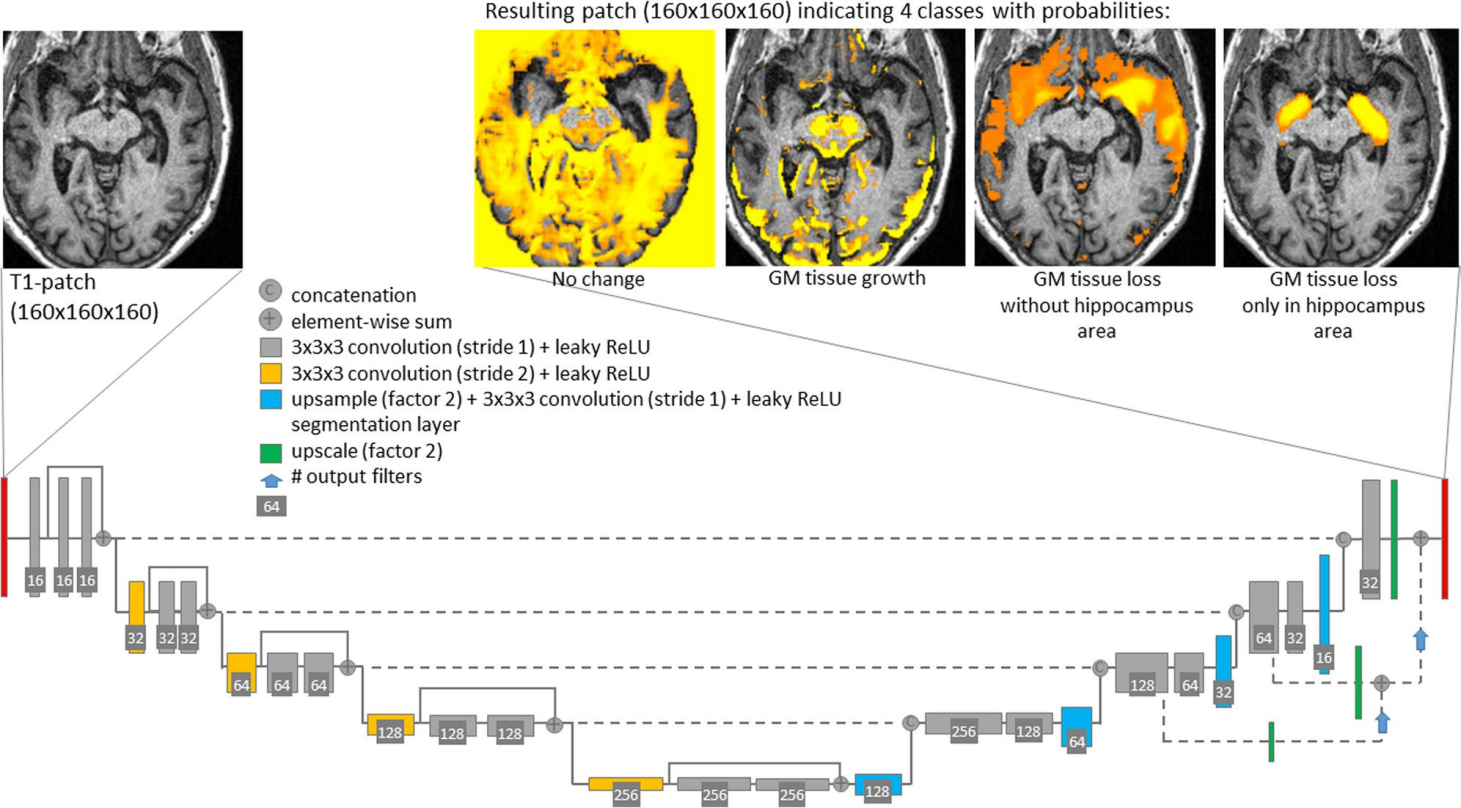
The proposed network for CNN-based single-subject VBM. The T1w-MRI scan is re-sampled to a 3D volume with cubic voxels of 1 mm edge length. The CNN operates patch-wise with a patch size of 160 × 160 × 160 voxels. It uses a fully convolutional encoder-decoder architecture with 3D convolutions, residual-block connections, and four reductions of the feature map size. The CNN generates the VBM map in four disjoint parts (GM, gray matter)

Second, each statistical map from scanner-specific VBM in the training dataset was disassembled into four disjoint parts (Fig. 1): “low significance,” original *t* value if − 2 ≤ *t* ≤ 2, otherwise zero; “high GM density,” original *t* value if *t* > 2, otherwise zero; “low extrahippocampal GM density,” original *t* value if *t* < − 2 and voxel outside the bilateral hippocampus, otherwise zero; “low hippocampal GM density,” original *t* value if *t* < − 2 and voxel in the bilateral hippocampus, otherwise zero. The hippocampus was segmented by an in-house 3D-CNN previously proposed for thalamus segmentation [32] and then validated for segmentation of the thalamus and the hippocampi simultaneously (unpublished). The rationale for disassembling the t-maps into 4 disjoint parts was based on initial experiments in which the whole t-map was learned at once. In these experiments, the region of the hippocampus was learned well, but other brain regions were neglected. This is in line with previous reports that sub-classification of “too large” background can stabilize CNN training [33].

Third, the individual T1w-MRI (input to the 3D-CNN) and the 4 parts of the corresponding t-map from scanner-specific VBM (output) were re-sampled to cubic voxels with 1 mm edge length.

Finally, the training data was divided into 6 partially overlapping subsets with respect to the subjects’ age: < 40 years (*n* = 1388), 30 years < age < 50 years (*n* = 2070), 40 years < age < 60 years (*n* = 2974), 50 years < age < 70 years (*n* = 3231), 60 years < age < 80 years (*n* = 3510), and > 70 years (*n* = 3035). A separate CNN was trained for each age range.

The custom 3D-CNN used in the current study follows a fully convolutional encoder-decoder (U-net-like) architecture (Fig. 1). It was trained for 100 epochs using the “Adaptive-Moment-Estimation” optimizer [34] with Dice loss function (sum of Dice similarity coefficient over the 4 output classes) and batch size 1. An exponentially decaying learning rate *α* = *α*_0_ (1 − *e*/100 )^0.9^ was used, where *e* denotes the current epoch. The start value of the learning rate was *α*_0_ = 0.0004.

More details of the network architecture, data augmentation during training, and application of the 3D-CNN are given in the supplementary material.

The 3D-CNN was applied to each of the 118 T1w-MRI in the test set. For the T1w-MRI of a patient with a given age, the CNN was selected for which the patient’s age was closest to the center of the corresponding age range.

### Conventional VBM with a multiple-scanner NDB

Conventional VBM was also performed with reference to a mixed NDB comprising T1w-MRI from 136 patients scanned for unspecific symptoms (headache, dizziness) on 136 different MRI scanners (multiple-scanner VBM; Table 1). None of the patients had a history of or currently ongoing neurological or psychiatric disease. All images were free of abnormalities beyond those expected for the patients’ age based on visual inspection by an experienced radiologist.

### Quantitative comparison and visual reading of VBM maps in the test set

Conventional VBM maps were thresholded at one-sided *p* = .005 (uncorrected for multiple comparisons), and CNN-VBM maps were thresholded at the corresponding cutoff 0.4 (“Application of the 3D-CNN” in the supplementary material), both for quantitative comparison and for visual reading. There was no threshold on the cluster size with any of the VBM methods.

Quantitative agreement of thresholded VBM maps from CNN-VBM or from multiple-scanner VBM with the gold standard maps from scanner-specific VBM was characterized using the Dice similarity coefficient.

**Fig. 2:**
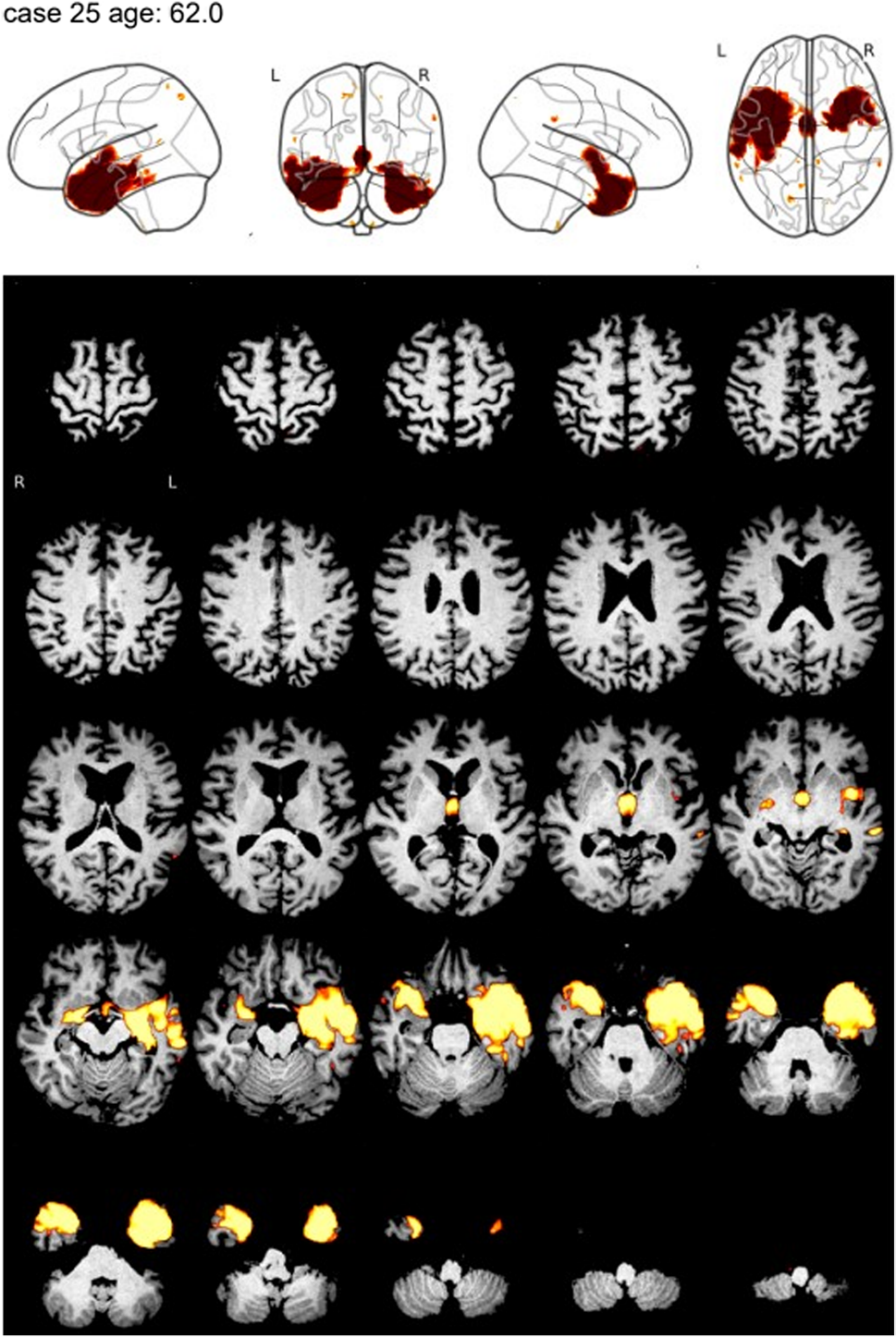
Standardized display for visual interpretation of the thresholded VBM map. The display combines transversal slices of the thresholded VBM map overlaid to the individual T1w-MRI scan and a glass brain view of the thresholded VBM map in a single-page pdf document. The example shows the CNN-VBM map of a patient with semantic variant primary progressive aphasia

For visual interpretation of the thresholded VBM maps, a standardized display in a single-page pdf document was used (Fig. 2). There were 354 different pdf documents: 118 test cases × 3 VBM methods. A copy was generated from each of these pdf documents to allow the assessment of intra-reader variability of the visual interpretation. The 708 pdf documents were presented in randomized order to two independent readers (DH, RB) with ≥ 7 years of experience in reading VBM maps in patients with suspected neurodegenerative disease. The readers were blinded for all clinical information except age.

The readers used a two-step approach for visual interpretation. First, the thresholded VBM map was categorized as indicative of “neurodegenerative disease,” “normal” (no neurodegenerative disease), or “uncertain.” In the second step, “neurodegenerative disease” cases were subcategorized as “mild cognitive impairment due to AD,” “AD dementia,” “posterior cortical atrophy,” “behavioral frontotemporal dementia,” or “semantic primary progressive aphasia”.

Intra-reader inconsistencies were resolved separately by each reader in a second reading session to obtain an intra-reader consensus. Between-readers inconsistencies were resolved in a joint reading session of the two readers to obtain a between-readers consensus.

The standardized display for visual reading did not include a colorbar, because voxel intensities in CNN-VBM maps cannot be directly interpreted as *p* values. The readers were asked to base their interpretation of the thresholded VBM maps on the localization/regional distribution pattern of significant clusters without taking their color into account.

### Statistical analysis

Cross tables and Cohen’s kappa were used to assess intra- and between-readers agreement of the visual interpretation and to assess the accuracy of the between-readers consensus relative to the clinical ground truth diagnoses, separately for each VBM method. The three AD subtypes were combined into one single AD category for this purpose, and the two FTLD subtypes were combined into a single FTLD category. “Uncertain” was considered a distinct category for the assessment of intra- and between-readers agreement. For the comparison with the ground truth diagnoses, the “uncertain” cases were included in the “normal” category.

The statistical analyses were repeated using a binary categorization: “any neurodegenerative disease” (AD or FTLD) versus “normal.”

## Results

**Fig. 3:**
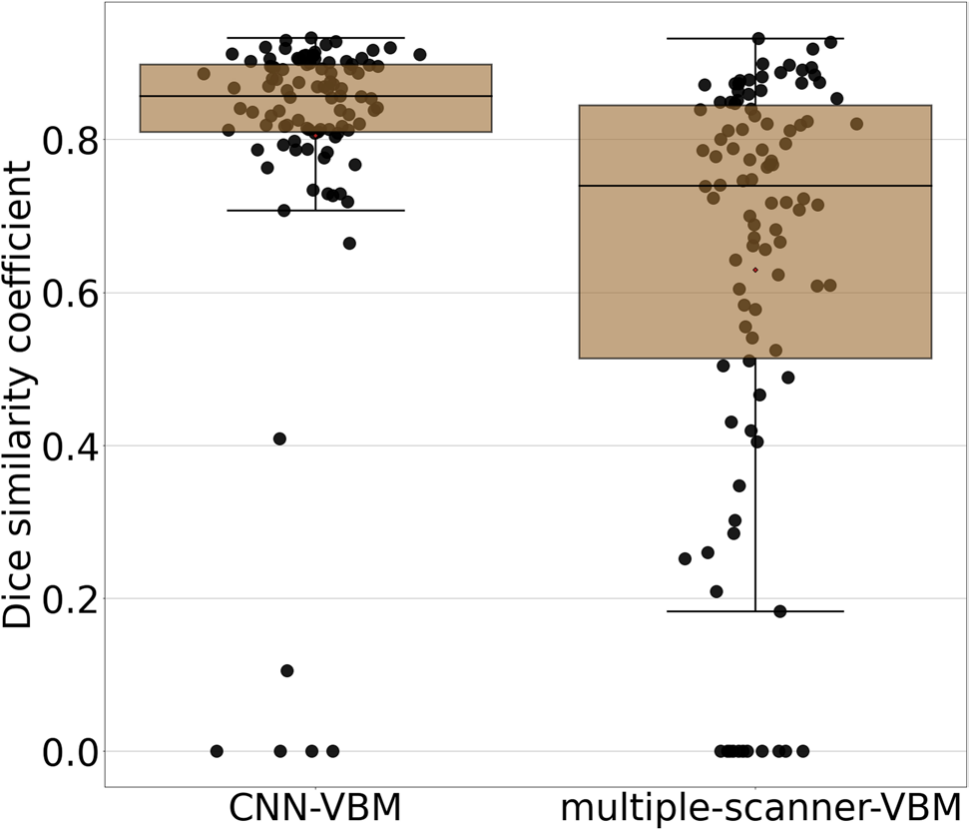
Quantitative comparison of the VBM maps in the independent test set: box-and-whisker plots of the Dice similarity coefficient between scanner-specific VBM and CNN-VBM (left) and between scanner-specific VBM and multiple-scanner VBM (right)

Box-and-whisker plots of the Dice similarity coefficient of CNN-VBM and multiple-scanner VBM maps relative to the scanner-specific VBM maps are shown in Fig. 3. The median Dice similarity coefficient relative to the gold standard maps was higher for CNN-VBM than for the multiple-scanner VBM (median 0.85, interquartile range [0.81, 0.90], versus 0.74 [0.51, 0.84], Wilcoxon’s signed-rank test *p* < .001).

Intra- and between-readers cross tables of the visual interpretation of the VBM maps are given in Supplementary Tables 1 and 2. The resulting kappa values are summarized in Table 2.

**Table 2:** Visual interpretation of VBM maps: Cohen kappa coefficients (and their 95%CI) for intra- and between-readers agreement and for the agreement between the consensus interpretation of the two readers with the ground truth diagnoses. The kappa values are given for the categorization according to three classes (AD versus FTLD versus normal) and for the detection of any neurodegenerative disease (AD or FTLD versus normal)

		Scanner-specific VBM	Multiple-scanner VBM	CNN-VBM
Intra-reader Reader 1	3 classes: AD vs FTLD vs normal	0.82 [0.73, 0.90]	0.79 [0.70, 0.89]	0.82 [0.73, 0.90]
	2 classes: AD or FTLD vs normal	0.84 [0.75, 0.94]	0.78 [0.67, 0.89]	0.79 [0.67, 0.90]
Intra-reader Reader 2	3 classes: AD vs FTLD vs normal	0.86 [0.78, 0.93]	0.94 [0.88, 1.00]	0.85 [0.78, 0.93]
	2 classes: AD or FTLD vs normal	0.88 [0.80, 0.96]	0.97 [0.93, 1.00]	0.84 [0.75, 0.93]
Between-reader	3 classes: AD vs FTLD vs normal	0.85 [0.77, 0.93]	0.84 [0.75, 0.93]	0.74 [0.64, 0.84]
	2 classes: AD or FTLD vs normal	0.88 [0.80, 0.96]	0.87 [0.79, 0.96]	0.71 [0.59, 0.83]
Reader consensus versus ground truth	3 classes: AD vs FTLD vs normal	0.77 [0.67, 0.87]	0.44 [0.32, 0.57]	0.72 [0.61, 0.82]
	2 classes: AD or FTLD vs normal	0.77 [0.65, 0.89]	0.37 [0.21, 0.52]	0.77 [0.65, 0.89]

*AD* Alzheimer’s disease, *CNN* convolutional neural network, *CNN-VBM* CNN-based VBM without reference to a normal database, *FTLD* frontotemporal lobar degeneration, *multiple-scanner VBM* conventional VBM with a mixed normal database comprising T1w-MRI images from multiple scanners as reference, *scanner-specific VBM* conventional VBM with a scanner- and sequence-specific normal database as reference, *VBM* voxel-based morphometry

Cross tables of the visual consensus interpretation of the VBM maps versus the ground truth diagnoses are given in Table 3 and Supplementary Table 3. The corresponding kappa values are given in Table 2. For the categorization according to three classes (AD versus FTLD versus normal), the kappa relative to the ground truth diagnoses was 0.77 for scanner-specific VBM (95%CI 0.67–0.87), 0.72 for CNN-VBM (0.61–0.82), and 0.44 for multiple-scanner VBM (0.32–0.57). For the detection of any neurodegenerative disease (AD or FTLD versus normal), the kappa relative to the ground truth diagnoses was 0.77 (0.65–0.89) for both the scanner-specific VBM and the CNN-VBM, and 0.37 (0.21–0.52) for the multiple-scanner VBM. Overall accuracy, sensitivity, and specificity of the consensus visual interpretation of the VBM maps for the detection of any neurodegenerative disease were 89.0% (105/118, 95%CI 81.9–94.0%), 84.0% (68/81, 74.1–91.2%), and 100% (37/37, 90.5–100%) for scanner-specific VBM; 89.8% (106/118, 82.9–94.6%), 90.1% (73/81, 81.5–95.6%), and 89.2% (33/37, 74.6%–97.0%) for CNN-VBM; and 64.4% (76/118, 55.1–73.0%), 48.1% (39/81, 36.9–59.5%), and 100% (37/37, 90.5–100%) for multiple-scanner VBM, respectively. The different VBM maps of a healthy subject incorrectly categorized as AD by the visual interpretation of the CNN-VBM map are shown in Supplementary Figure 2. The VBM maps of an AD patient incorrectly categorized as normal by the visual interpretation of the multiple-scanner VBM map are shown in Supplementary Figure 3.

**Table 3:** Visual interpretation of VBM maps: cross tables of the reader consensus versus the ground truth diagnoses for the differentiation between AD, FTLD, and normal, separately for each of the three different VBM methods

		Scanner-specific VBM			Multiple-scanner VBM			CNN-VBM		
		AD	FTLD	Normal	AD	FTLD	Normal	AD	FTLD	Normal
Ground truth	AD	38	2	11	13	2	36	38	7	6
	FTLD	3	25	2	1	23	6	3	25	2
	Normal	0	0	37	0	0	37	3	1	33

*AD* Alzheimer’s disease, *CNN* convolutional neural network, *CNN-VBM* CNN-based VBM without reference to a normal database, *FTLD* frontotemporal lobar degeneration, *multiple-scanner VBM* conventional VBM with a mixed normal database comprising T1w-MRI images from multiple scanners as reference, *scanner-specific VBM* conventional VBM with a scanner- and sequence-specific normal database as reference, *VBM* voxel-based morphometry

## Discussion

Conventional single-subject VBM to support the diagnosis of neurodegenerative diseases is restricted by the need of a scanner- and sequence-specific NDB. To overcome this barrier, a 3D-CNN for single-subject VBM was trained on a large, multi-site dataset of T1w-MRI. In an independent test set, CNN-based VBM achieved a similar performance as the gold standard VBM with a scanner- and sequence-specific NDB both on a technical level and with respect to clinical utility. Thus, CNN-VBM eliminates the need for “expensive” NDB and, therefore, can pave the way for the widespread use of single-subject VBM in clinical routine. To the best of our knowledge, this is the first study to test a method for single-subject VBM without a NDB.

Visual interpretation of CNN-VBM maps resulted in balanced sensitivity and specificity whereas visual interpretation of the scanner-specific VBM maps achieved perfect specificity at somewhat lower sensitivity. There were a few outliers with dissimilar VBM maps (Fig. 3). Some outliers were due to false-positive atrophy clusters by CNN-VBM (Supplementary Figure 2) that resulted in false-positive visual interpretation of the CNN-VBM map. Other outliers were due to the lack of atrophy clusters in CNN-VBM that were shown by scanner-specific VBM. In some of these cases, the CNN-VBM map was correctly interpreted as normal whereas the visual interpretation of the gold standard VBM map resulted in a false-positive diagnosis. Methods to improve the specificity of CNN-VBM will be tested in further studies.

While intra-reader agreement in the visual interpretation of the statistical VBM maps was substantial to almost perfect for all VBM methods, between-readers agreement was lower for CNN-VBM compared to that for scanner-specific VBM. This was mainly driven by a varying fraction of “uncertain” VBM maps according to reader 2 between CNN-VBM and scanner-specific VBM (15.3%, 18/118 versus 12.7%, 15/118), whereas the fraction of “uncertain” maps according to reader 1 was the same for both VBM methods (9.3%, 11/118). Retrospective consultation revealed that reader 2 used a more conservative approach for visual interpretation whereas reader 1 used a more sensitive approach. We hypothesize that between-readers agreement can be improved by a threshold on the cluster size in the VBM maps.

VBM maps from multiple-scanner VBM showed lower similarity with the scanner-specific VBM maps than CNN-VBM maps with larger between-subject variability (Fig. 3). This resulted in considerably lower overall accuracy of the visual interpretation of the multiple-scanner VBM maps for detection of AD or FTLD. The loss of accuracy was entirely driven by loss of sensitivity. Thus, multiple-scanner VBM might be useful for enrichment of datasets with MRI scans from patients with a neurodegenerative disease in research settings. It appears less useful for single-subject VBM in clinical routine when high sensitivity is required, too.

The 3D-CNN was trained with the full t-map from scanner-specific VBM without any threshold. This allows to operate the CNN at different significance levels without the need for re-training, similar to conventional VBM.

The utility of single-subject VBM has been questioned due to a rather high rate of false-positive findings associated with normal variability of single subjects’ neuroanatomy [20, 35]. However, the use of VBM to support the diagnosis of neurodegenerative diseases is based on the detection of disease-characteristic atrophy patterns that often comprise a rather large network of non-neighboring brain regions (in AD medial temporal lobe, temporoparietal junction, and posterior cingulate cortex). The false-positive findings in single-subject VBM often consist of rather small clusters in non-disease-specific brain regions and, therefore, might not be misinterpreted as indication of a neurodegenerative disease by an experienced reader.

A major strength of (CNN-)VBM is that it is not restricted to a predefined set of diseases, in contrast to support vector machines or other classifiers that have been trained for automatic categorization of MRI scans into a predefined set of disease classes [36–38]. VBM allows the detection of altered brain tissue concentration throughout the whole brain without a priori hypothesis about the localization. This is an advantage in clinical routine, in which a considerable fraction of patients is referred to brain MRI without a specific etiological pre-scan diagnosis.

It should be noted in this context that the clinical MRI scans in the multi-site dataset used for the network training had been acquired for a large variety of indications, not restricted to suspicion of AD or FTLD. This suggests that the network might be useful also in other diseases. This has to be tested in additional studies.

A limitation of the current study is that the scanner- and sequence-specific NDB used to generate the gold standard t-maps for CNN training comprised brain MRI scans that had been acquired for unspecific symptoms such as headache or dizziness. All images were free of abnormalities beyond those expected for the patients’ age based on visual inspection by an experienced radiologist. It cannot be ruled out that some patients had a neurodegenerative disease at an early stage without noticeable symptoms but mild disease-related atrophy that was not detected on visual inspection. This might have resulted in reduced sensitivity of the CNN-VBM for detection of atrophy, particularly for the detection of AD-typical atrophy in the older subjects, since asymptomatic AD probably is rather frequent at ≥ 70 years of age [39]. Furthermore, the inclusion of the T1w-MRI scans of the normal database for the test dataset in the test dataset might have caused some bias. However, the bias most likely was small and to the disadvantage of the CNN-based VBM (overestimation of the performance of conventional VBM would result in underestimation of the performance of CNN-VBM compared to conventional VBM as benchmark).

In conclusion, CNN-based single-subject VBM can provide a similar performance in the detection of AD- and FTLD-specific atrophy in brain T1w-MRI as the gold standard single-subject VBM with reference to a scanner- and sequence-specific NDB. This could pave the way for widespread use of VBM in everyday clinical routine, as the CNN estimates the VBM maps directly from the original MRI without reference to a NDB. The need of a scanner- and sequence-specific NDB is currently a major barrier for the routine use of single-subject VBM at many sites.

### Supplementary Information

Below is the link to the electronic supplementary material.

Supplementary file1 (PDF 721 KB)

## Abbreviations

3D: 3-dimensional
AD: Alzheimer’s disease
CNN: Convolutional neural network
CNN-VBM: CNN-based VBM without reference to a normal database
FTLD: Frontotemporal lobar degeneration
GM: Gray matter
Multiple-scanner VBM: Conventional voxel-based morphometry with a mixed normal database comprising T1w-MRI images from multiple scanners as reference
NDB: Normal database
Scanner-specific VBM: Conventional VBM with a scanner- and sequence-specific normal database as reference
VBM: Voxel-based morphometry

## Declarations

### Guarantor

The scientific guarantor of this publication is Ralph Buchert.

### Statistics and biometry

No complex statistical methods were necessary for this paper.

### Study subjects or cohorts overlap

The test dataset comprising T1w-MRI from 118 subjects was reported previously [22]. This prior article dealt with the utility of conventional voxel-based morphometry with reference to a scanner- and sequence-specific normal database to support the differential diagnosis of dementing neurodegenerative diseases whereas the current manuscript deals with the design, training, and testing of a convolutional neural network for voxel-based morphometry without a normal database. There is no further overlap with previously published studies or work currently undergoing review or in press at a journal. Neither the training dataset of 8945 MRI scans nor the convolutional neural network was described previously.

### Methodology

• retrospective

• diagnostic or prognostic study

• multi-center study
