# Supplementary material for: Voxel-based morphometry in single subjects without a scanner-specific normal database using a convolutional neural network
Source: Eur Radiol. 2023 Nov 9;34(6):3578–87. doi: 10.1007/s00330-023-10356-1 (PMC11166757; doi:10.1007/s00330-023-10356-1)
Supplement: Supplementary file 1 — Supplementary file1 (PDF 721 KB) [file 330_2023_10356_MOESM1_ESM.pdf]

Voxel-based morphometry in single subjects without scanner-specific normal database using a convolutional neural network

Electronic Supplementary Material

Conventional single-subject VBM

Conventional single-subject VBM was performed with the SPM12 software package (www.fil.ion.ucl.ac.uk/spm/). The processing steps are illustrated in Supplementary Figure 1. In brief, the original 3D T1w-MRI scan was segmented into a gray matter (GM), white matter, and cerebrospinal fluid component images. Spatial correspondence between the GM component image of the patient and the GM component images of the NDB was established via high dimensional non-linear image registration (DARTEL) [1]. The registered and modulated individual GM component image was smoothed by convolution with an isotropic Gaussian kernel of 8 mm full-width-at-half-maximum. After smoothing, a voxel-based two-sample t-test of the individual smoothed GM component image against the GM component images of the NDB was carried out, resulting in a statistical t-map. Age and total intracranial volume were taken into account as nuisance covariates. The total intracranial volume was estimated in each T1w-MRI scan by using a 3D-CNN specifically trained for accurate and stable delineation of the total intracranial volume [2; 3].

3D-CNN architecture

The architecture of the custom 3D-CNN is shown in Figure 1 in the manuscript. The 3D-CNN follows a fully convolutional encoder-decoder (U-net-like) architecture with 3D convolutions with 3x3x3 kernel size. Residual blocks are used in the encoder [4]. In addition, deep supervision [5] is employed by including additional segmentation layers at several stages in the decoder. The encoder reduces the spatial feature map size four times (using convolution with stride 2) and doubles the feature map number with each reduction. Starting with 16 feature maps of size 160x160x160 in the first layer, this leads to

256 maps of size 10x10x10 in the last encoder layer. The decoder uses convolution layers, followed by nearest-neighbour up-sampling and deep supervision in three layers [5]. For the long-range connections between encoder and decoder a feature concatenation is employed. Leaky ReLU [6] is used as activation function in each layer. Due to the rather large patches of 160x160x160 voxels the batch size is 1. Therefore, instance normalization, a special case of group normalization, is used instead of batch normalization.

Data augmentation

Random combinations of the following data augmentation techniques were used during training of the 3D-CNN to further increase the heterogeneity of the training dataset.

- Random left-right flipping of the image patches.
- Rotation around the x-, y- or z-axis by an angle randomly chosen between -10 and 10 degrees.
- Translation in x-, y- or z-direction by a distance randomly chosen between -5 and 5 mm.
- Voxel-wise adding of Gaussian random noise with zero mean and variance randomly chosen between 0 and 0.0001. The voxel values were normalized between 0 and 1 (this is required for every CNN)
- Simulation of a random bias field. A plane was selected randomly within the 3D volume. For all other planes, the distance to the selected plane was computed. Distance values were scaled to the interval [1, d] with d a random number between 1 and 2. Image voxel values were multiplied with the scaled distance of the plane the voxel was located in.

The first three augmentation techniques were applied simultaneously to the input and to the output of the 3D-CNN whereas the last two techniques were applied only to the input image (output unchanged).

Application of the 3D-CNN

For the application of the 3D-CNN to a T1w-MRI scan, eight (2x2x2) evenly distributed overlapping crops of 160x160x160 mm^3 were taken. For each crop, the predicted class values were computed and

merged to the entire volume by taking the mean values in the overlapping regions. Each of the 4 output maps (corresponding to the 4 parts of the statistical maps from scanner-specific-VBM, Figure 1 in the manuscript) contains (probability) values between 0 and 1. The output maps corresponding to the 'low significance' part and to the 'high GM density' part of conventional t-maps were ignored. The output maps corresponding to the 'low extrahippocampal GM density' part and to the 'low hippocampal GM density' part were summed voxel-by-voxel to obtain the (final) CNN-VBM map.

The voxel intensities in the CNN-VBM map range between 0 and 1. The threshold corresponding to P=.005 for t-maps from scanner-specific-VBM was identified as follows. On a subset of 130 randomly selected cases from the training dataset (2 cases per scanner), the CNN-VBM-map was binarized using varying thresholds between 0.1 and 0.85 (step size 0.05). For each threshold on the CNN-VBM-map, the Dice similarity coefficient of the resulting binarized map with the corresponding conventional gold standard map from scanner-specific-VBM binarized at P=.005 was computed for each of the 130 cases. Prior to computing the Dice similarity coefficient, the binary maps were smoothed by convolution with an isotropic Gaussian kernel with 10 mm full-width-at-half-maximum and then binarized again (all values > 0) in order to reduce the sensitivity with respect to minor, clinically irrelevant differences. The mean Dice similarity coefficient over the 130 cases was maximal for a threshold of 0.40 (Supplementary Figure 4). This value was used for thresholding the CNN-VBM-maps for visual interpretation.

References to the supplementary material

1 Ashburner J (2007) A fast diffeomorphic image registration algorithm. Neuroimage 38:95-113

2 Adduru VR, Michael AM, Helguera M, Baum SA, Moore GJ (2017) Leveraging Clinical Imaging Archives for Radiomics: Reliability of Automated Methods for Brain Volume Measurement. Radiology 284:862-869

3 Opfer R, Kruger J, Spies L, Kitzler HH, Schippling S, Buchert R (2022) Single-subject analysis of regional brain volumetric measures can be strongly influenced by the method for head size adjustment. Neuroradiology. 10.1007/s00234-022-02961-6

4 He K, Zhang X, Ren S, Sun J (2016) Deep residual learning for image recognitionProceedings of the IEEE conference on computer vision and pattern recognition, pp 770-778

5 Dou Q, Yu L, Chen H et al (2017) 3D deeply supervised network for automated segmentation of volumetric medical images. Medical image analysis 41:40-54

6 Maas AL, Hannun AY, Ng AY (2013) Rectifier nonlinearities improve neural network acoustic models. Proc. icml 30 (1), 3

Supplementary Tables

Supplementary Table 1. Visual interpretation of VBM-maps: intra-reader cross tables

		scanner-specific-VBM				multiple-scanner-VBM				CNN-VBM			
		reader 1 vote 2				reader 1 vote 2				reader 1 vote 2			
		AD	FTLD	normal	uncert	AD	FTLD	normal	uncert	AD	FTLD	normal	uncert
reader 1 vote 1	AD	40	1	1	5	17	1	1	2	46	1	0	2
	FTLD	4	23	0	0	0	24	0	0	2	29	0	0
	normal	0	0	34	2	3	0	60	2	1	0	21	6
	uncert	0	0	2	6	1	1	4	2	0	2	1	7
		reader 2 vote 2				reader 2 vote 2				reader 2 vote 2			
reader 2 vote 1	AD	35	2	0	0	10	2	0	0	36	3	1	0
	FTLD	3	25	0	0	1	22	0	0	1	29	0	0
	normal	0	0	36	2	0	0	70	2	2	0	24	4
	uncert	4	0	2	9	4	1	1	5	7	1	3	7

(**AD**: Alzheimer's disease, **CNN**: convolutional neural network, **CNN-VBM**: CNN-based VBM without reference to a normal database, **FTLD**: frontotemporal lobar degeneration, **multiple-scanner-VBM**: conventional VBM with a mixed normal datadase comprising T1w-MRI images from multiple scanners as reference, **scanner-specific-VBM**: conventional VBM with a scanner- and sequence-specific normal database as reference, **uncert**: uncertain, **VBM**: voxel-based morphometry)

Supplementary Table 2. Visual interpretation of VBM-maps: between-reader cross tables

		scanner-specific-VBM				multiple-scanner-VBM				CNN-VBM			
		reader 1				reader 1				reader 1			
		AD	FTLD	normal	uncert	AD	FTLD	normal	uncert	AD	FTLD	normal	uncert
reader 2	AD	35	2	0	0	10	2	0	0	36	3	1	0
	FTLD	3	25	0	0	1	22	0	0	1	29	0	0
	normal	0	0	36	2	0	0	70	2	2	0	24	4
	uncert.	4	0	2	9	4	1	1	5	7	1	3	7

(**AD**: Alzheimer's disease, **CNN**: convolutional neural network, **CNN-VBM**: CNN-based VBM without reference to a normal database, **FTLD**: frontotemporal lobar degeneration, **multiple-scanner-VBM**: conventional VBM with a mixed normal datadase comprising T1w-MRI images from multiple scanners as reference, **scanner-specific-VBM**: conventional VBM with a scanner- and sequence-specific normal database as reference, **uncert**: uncertain, **VBM**: voxel-based morphometry)

Supplementary Table 3. Visual interpretation of VBM-maps: cross tables of the reader consensus versus the ground truth diagnoses for the detection of any neurodegenerative disease (AD or FTLD versus normal), separately for each of the three different VBM methods

		scanner-specific-VBM		multiple-scanner-VBM		CNN-VBM	
		AD/FTLD	normal	AD/FTLD	normal	AD/FTLD	normal
ground truth	AD/FTLD	68	13	39	42	73	8
	normal	0	37	0	37	4	33
sens/spec/PPP/NPV		0.84/1.00/1.00/0.74		0.48/1.00/1.00/0.47		0.90/0.89/0.95/0.80	

(**AD**: Alzheimer's disease, **CNN**: convolutional neural network, **CNN-VBM**: CNN-based VBM without reference to a normal database, **FTLD**: frontotemporal lobar degeneration, **multiple-scanner-VBM**: conventional VBM with a mixed normal datadase comprising T1w-MRI images from multiple scanners as reference, **NPV**: negative predictive value, **PPV**: positive predictive value, **scanner-specific-VBM**: conventional VBM with a scanner- and sequence-specific normal database as reference, **sens**: sensitivity, **spec**: specificity, **VBM**: voxel-based morphometry)

Supplementary Figures

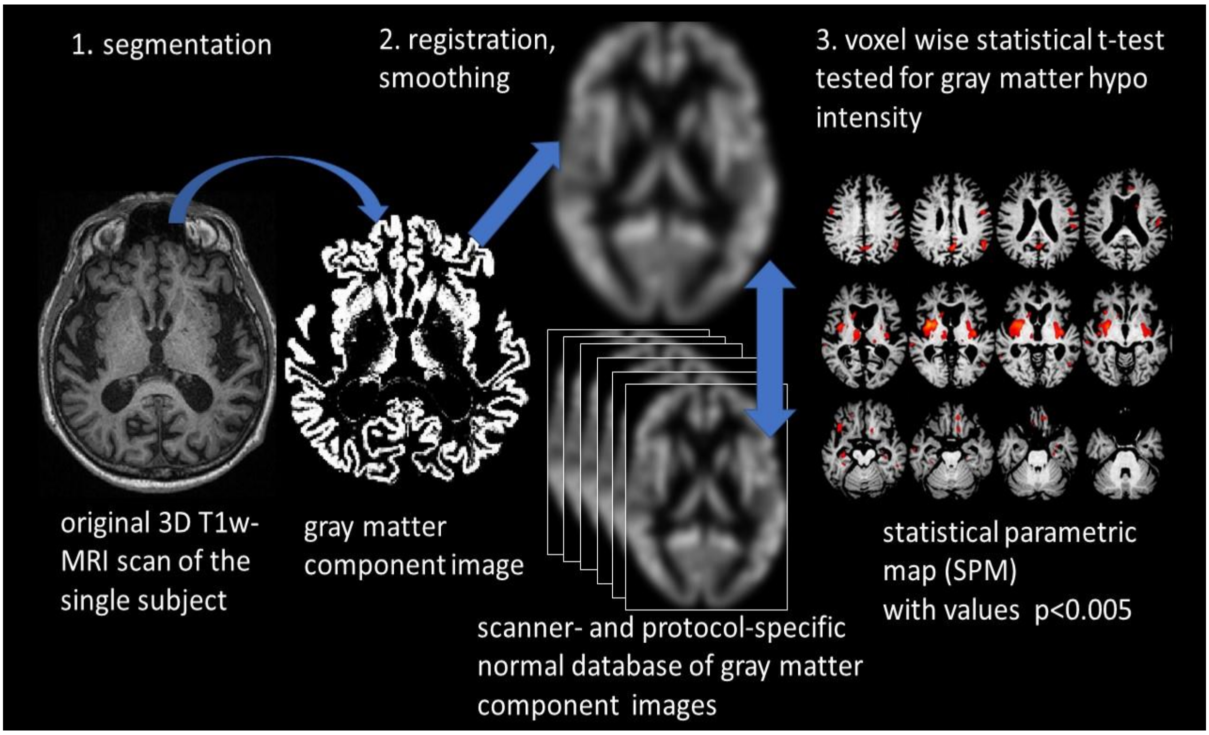

Supplementary Figure 1. Conventional voxel-based morphometry with reference to a scanner- and protocol-specific normal database.

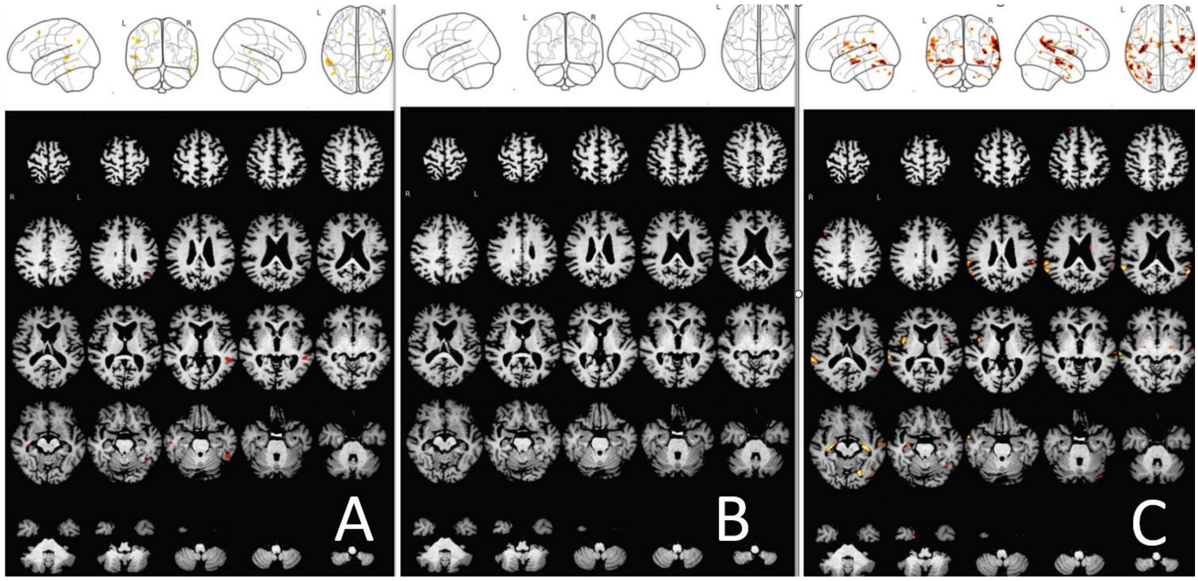

Supplementary Figure 2. VBM-maps of one of the four false positive cases according to the visual consensus interpretation of the CNN-VBM-map. The CNN-VBM-map of the 73y old healthy control subject (C) was incorrectly categorized as AD whereas the gold standard map from scanner-specific-VBM (A) and the map from multiple-scanner-VBM (B) were correctly classified as normal.

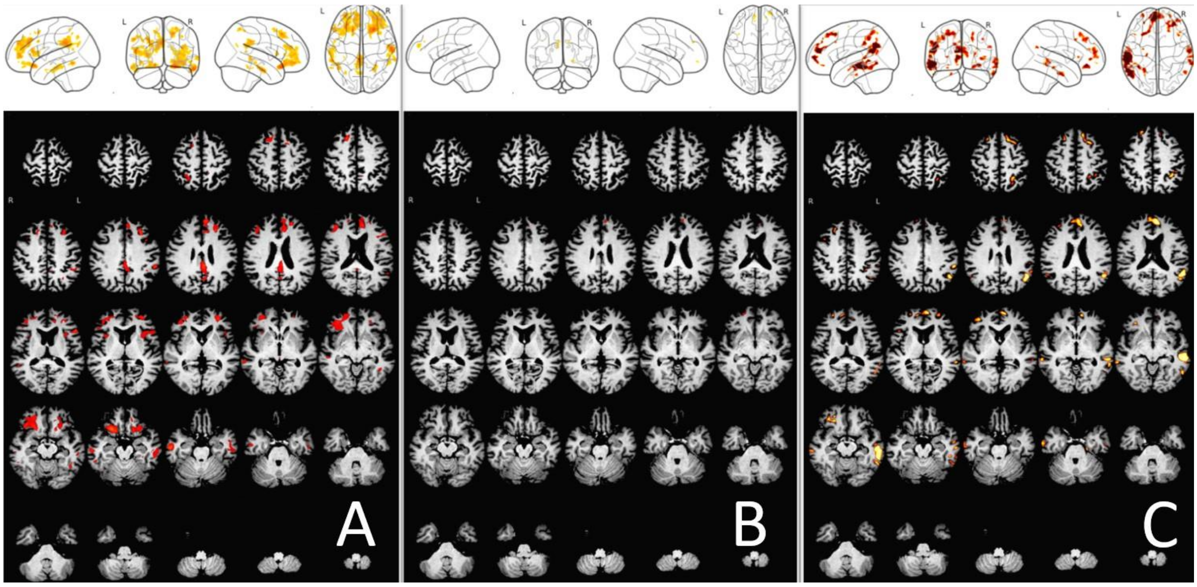

Supplementary Figure 3. VBM-maps of one of the 42 false negative according to multiple-scanner-VBM (B). The CNN-VBM-map of the 73y old AD patient (C) was categorized correctly, the gold-standard map from scanner-specific-VBM (A) was misclassified as FTD.

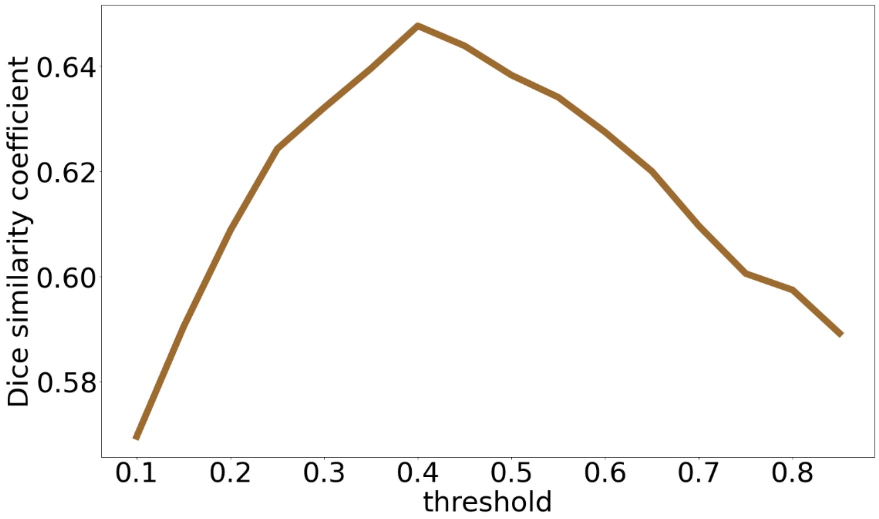

Supplementary Figure 4. Mean Dice similarity coefficient between the thresholded CNN output with the gold standard t-maps from scanner-specific-VBM thresholded at P=.005 for varying thresholds on the CNN output.
